# Enhanced Written vs. Verbal Recall Accuracy Associated With Greater Prefrontal Activation: A Near-Infrared Spectroscopy Study

**DOI:** 10.3389/fnbeh.2021.601698

**Published:** 2021-03-30

**Authors:** Jianan Zhang, Ya Wang, Yu Zhang, Brian Li, Yi Zhang

**Affiliations:** ^1^Department of Rehabilitation Medicine, Third Affiliated Hospital of Soochow University, Changzhou, China; ^2^Princeton University, Princeton, NJ, United States

**Keywords:** memory, short-term memory, near-infrared spectroscopy, NIRS, verbal, medial prefrontal cortex

## Abstract

**Background:** Memory efficiency is influenced by the modalities of acquisition and retrieval. The recall accuracy of read or voiced material differs depending on whether the recall is given verbally or in writing. The medial prefrontal cortex (mPFC) is critical for both attentional allocation and short-term memory, suggesting that different short-term memory recall modalities are associated with distinct mPFC processes and activation patterns.

**Methods:** Near-infrared spectroscopy (NIRS) was used to monitor mPFC oxygenation parameters of 30 healthy subjects during acquisition and recall tasks as a measure of neural activity. Oxygenation parameters and recall accuracy were compared between oral and written answers and the potential correlations were analyzed.

**Results:** Written responses were more accurate than verbal responses to the same questions and evoked greater changes in mPFC oxyhemoglobin (oxyHb) and total Hb (total-Hb). Furthermore, there were significant positive correlations between recall accuracy and both Δ[oxyHb] and Δ[total-Hb] in the mPFC.

**Conclusion:** Memory accuracy of written material is greater when responses are also written rather than verbal. In both cases, recall accuracy was correlated with the degree of mPFC activity. This NIRS-based learning and memory paradigm may be useful for monitoring training efficacy, such as in patients with cognitive impairment.

## Introduction

Learning and memory involve are multi-step neural which include the encoding and acquisition of information (memorization), its storage and retention (consolidation), and finally its reproduction or recognition (recall) (Amodio, 2019; Fukuda et al., 2020). Temporal patterns of presentation (spaced or condensed) and the modality of information presentation and recall can affect overall performance, which further adds to the complexity of these processes. However, few studies have examined the effect of output modality on memory performance for the same input material and the associated differences in underlying neural processing patterns. For instance, performance and spatiotemporal neural activity during recall of written information (acquired by reading) may differ depending on whether recall is rewritten or verbal.

The prefrontal cortex (PFC) contributes to numerous advanced brain functions (D'Esposito et al., 2000; Davidson et al., 2006). Both the lateral and medial surfaces of the PFC receive projections from medial temporal lobe (MTL) and thalamus, which are part of the recognition memory network (Banks et al., 2012; Pergola and Suchan, 2013; Preston and Eichenbaum, 2013; Guo and Yang, 2019) which are critical for recall (Cohen et al., 2018). Medial prefrontal cortex (mPFC) plays a critical role in remote, recent and short-term memories over a broad range of tasks (Euston et al., 2012). Different recall modalities may result in different levels of mPFC activity.

These mPFC functions have been revealed by analyzing associations between regional brain injury and functional deficits, as well as by multiple neuroimaging modalities during tasks that engage various cognitive functions. Such neuroimaging studies have found that mPFC is active during the maintenance and manipulation of information in working memory (Nee et al., 2013), cognitive control, and attentional regulation (Rowe et al., 2000; Miller and Cohen, 2001; Lissek and Gunturkun, 2004). Near-infrared spectroscopy (NIRS) allows for acquisition and analysis of such phenomena in real time. Increased regional brain activity is associated with greater local metabolism and blood flow, and NIRS provides a method for measuring neural activity associated with local changes in hemoglobin (Hb) concentration (Miller and Cohen, 2001). By exploiting the different optical absorption curves of oxyhemoglobin (oxyHb) and deoxyhemoglobin (deoxyHb), NIRS can monitor tissue metabolism and brain activity (la Cour et al., 2018) with accuracy comparable to functional magnetic resonance imaging (fMRI) (Moriguchi et al., 2017). However, unlike fMRI, NIRS is portable, non-invasive, places fewer behavioral constraints on subjects, and is much less costly (Yeung et al., 2016a). Moreover, NIRS can be used for some patients with contraindications to fMRI. These advantages have served to greatly expand the use of NIRS for studying human brain function (Weigl et al., 2016; Agbangla et al., 2017; Annavarapu et al., 2019).

This study used a two-channel NIRS system to monitor changes in oxyHb and deoxyHb concentrations in bilateral mPFC during the written or verbal recall of read material. The primary objective of this study was to examine how output modality affects memory performance and underlying mPFC activity. Specifically, this study assessed (1) whether mPFC Hb parameters differ during written and verbal responses, (2) whether memory accuracy differs according to recall modality, and (3) if there is a correlation between Hb changes in mPFC and recall accuracy.

## Methods

### Subjects

Thirty right-handed subjects aged 22–30 years (16 males, 14 females) with an average of 16.27 ± 0.59 years of formal education were recruited. The sleep time before the experiment was more than 7 h in all cases (7.48 ± 0.85 h) (Cespedes et al., 2016). None of the participants reported alcohol intake or drug use on the day of the experiment and no participant reported a history of neurological or psychiatric disorders. All participants had normal or corrected-to-normal vision. Each participant provided informed written consent, and the study protocol was approved by the Ethics Committee of the Third Affiliated Hospital of Soochow University.

### Equipment

**Figure 1:**
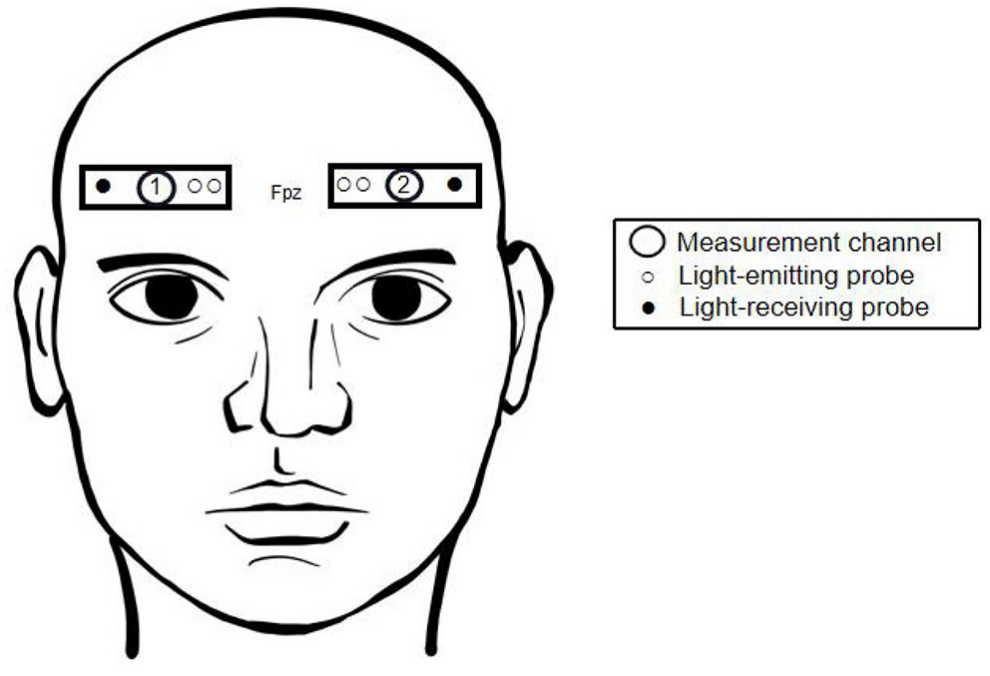
Two measurement channels centered at Fpz.

We used a dual-channel NIRS system (EGOS-600A; Enginmed Bio-Medical Electronics, Suzhou, China) to measure hemodynamic responses from the foreheads of all 30 subjects. According to the international 10–20 electrode system, two pairs of light detectors were placed at positions Fp1-F7 and Fp2-F8, and corresponding light sources were placed at Fp1 and Fp2, 3 and 4 cm from the two detectors, respectively. Based on previous studies (Okamoto et al., 2004; Koessler et al., 2009), it was estimated that the channels were located around the mPFC. In this configuration, tissue Δ[oxyHb] and Δ[deoxyHb] can be detected at a depth of 2–3 cm below the scalp surface (Figure 1). The device uses three near-infrared wavelengths, 760, 810, and 840 nm, and calculates Δ[oxyHb] and Δ[deoxyHb] according to the modified Beer-Lambert law. Total hemoglobin concentration (Δ[total-Hb]) was then calculated as the sum of the two. Output was sampled every 2 s and both high-pass and low-pass filtered to reduce noise interference. During the experiment, subjects were asked to minimize head shaking to reduce error caused by changes in hydrostatic pressure.

### Experimental Protocol

The experiment was conducted in a 3 × 4 m room, and the air conditioner was adjusted to maintain the room temperature at 20–24°C, which reduced changes in instrument sensitivity due to forehead perspiration. The NIRS probes were fixed to the subject after wiping the skin surface with an alcohol-soaked cotton ball (to reduce effects of oil, cosmetics, etc.) and organizing the subject's hair. The exterior of the probe was fixed to the forehead by a bandage to reduce natural light interference and prevent the probe from falling off during the experiment.

After the probe was fixed, the subject rested while cerebral oxygen parameter recording began. During the experiment, the subjects read two articles (Su, 2005; Zhang and Sun, 2018), each about 3,000 words in length. The reading time was 10 min per article. Subjects were then questioned verbally on the contents of the article and responded either in writing or verbally. For example, after reading an article on a societal issue, the subject was asked about the potential solutions provided by the article's author. If incorrect answers were provided, the subjects were allowed to correct themselves, whether in writing or verbally. The experimenter scored the answers from 0 to 10 according to a unified rubric, where points were earned for correctly identifying or summarizing the content of the article in accordance with the provided questions. There was a 10-min break between the two reading and question sessions. To reduce priming, the subjects were randomly assigned to two groups. Group A provided oral responses to oral questions after reading the first article and completed written answers to oral questions after reading the second article, while Group B read articles in the same order but provided written responses to questions on the first article while providing oral responses to questions about the second. In addition, the first 10 s of each recording period was removed to reduce the residual effects of the previous task. Based on previous studies on comparable subjects, we found that most oral responses could be provided within 5 min, while written answers could be provided within 10 min. As such, to reduce experimental error, we limited oral response times to 5 min and written response times to 10 min. After reading each article and providing answers, subjects scored the difficulty of the reading material and questions from 0 to 10. The responses of all subjects were then scored by two researchers, and the average score was recorded.

### Statistical Analysis

Average Δ[oxyHb] and Δ[deoxyHb] values were calculated for each subject at pre-task baseline and during both reading and answering sessions. Shapiro–Wilk W tests were used to check normality for each continuous variable. Values were then compared according to answer type for the same article (written vs. oral). We also examined the correlations between NIRS measurements and answer scores.

*T*-tests were used to compare measured differences in brain oxygen parameters at different points in the experiment. Pearson correlation analyses and descriptive statistical analysis methods were then used. All analyses were performed using SPSS 26.0 software, where a statistical significance was set at *p* < 0.05.

## Results

### Behavioral Data

All 30 subjects completed the experiment, but NIRS data from four male subjects were removed due to changes in probe sensitivity caused by forehead perspiration. The self-rated difficulty scores were moderate for both articles (4.85 ± 1.51 and 4.77 ± 1.37, respectively). Tables 1, 2 summarizes basic demographic information of the participants and the subjects' scores for oral and written answers. Scores for written answers were higher than scores for oral answers for both articles.

**Table 1:** Demographic characteristics of subjects.

**Group**	**Number**	**Sex (Male/Female)**	**Age (years)**	**Education (years)**	**Sleep duration (hours)**
A	14	7/7	25.14 (2.23)	16.29 (0.59)	7.49 (0.97)
B	12	5/7	25.17 (1.82)	16.25 (0.60)	7.47 (0.67)
Total	26	12/14	25.15 (2.05)	16.27 (0.59)	7.48 (0.85)

*Age, education, and sleep duration expressed as mean (standard deviation)*.

**Table 2:** Demographic characteristics of participants and article test scores.

	**Group A**		**Group B**		**Total**		
	**Mean**	**SD**	**Mean**	**SD**	**Mean**	**SD**	
Number	14		12		26		
Sex (M/F)	7/7		5/7		12/14		
Age (years)	25.14	2.23	25.17	1.82	25.15	2.05	
Education (years)	16.29	0.59	16.25	0.60	16.27	0.59	
Sleep duration (hours)	7.49	0.97	7.47	0.67	7.48	0.85	
	**Oral answers**		**Written answers**		***P***	**Total**	
	**Mean**	**SD**	**Mean**	**SD**		**Mean**	**SD**
Article 1 (score)	5.857	0.819	6.792	0.582	0.003^**^	6.288	0.851
Article 2 (score)	5.917	0.634	7.036	0.664	<0.001^**^	6.519	0.854
*P*	0.840		0.333		—	0.334	

** *P < 0.01*.

### NIRS Results

Both [oxyHb] and [total-Hb] increased significantly while subjects read and answered questions. There were no significant differences in cerebral oxygen parameters between the two reading sessions for subjects (Table 3), indicating a small or absent priming effect, possibly due to the intervening 10-min rest period. However, Δ[oxyHb] and Δ[total-Hb] were significantly higher for written answers than verbal answers to the same question (always presented verbally) on the same article (Table 4). Moreover, Δ[oxyHb] and Δ[total-Hb] were greater for written answers compared to verbal answers in response to different questions (Table 3).

**Table 3:** Cerebral oxygen parameters of 26 subjects during article reading.

		**Δ[deoxy-Hb]**	**Δ[oxy-Hb]**	**Δ[t-Hb]**
Left forehead	Article 1	0.020 (0.944)	1.970 (3.110)	1.990 (3.414)
	Article 2	0.330 (0.934)	1.230 (3.487)	1.559 (3.686)
	*P*	0.240	0.423	0.664
Right forehead	Article 1	0.001 (1.024)	2.123 (3.299)	2.124 (3.702)
	Article 2	0.281 (1.038)	2.145 (3.558)	2.424 (4.016)
	*P*	0.331	0.982	0.781
Full forehead	Article 1	0.011 (0.827)	2.047 (2.613)	2.057 (3.043)
	Article 2	0.305 (0.888)	1.687 (3.328)	1.991 (3.705)
	*P*	0.585	0.667	0.945

**Table 4:** Comparison of cerebral oxygen parameters between oral recall and write recall.

		**Δ[deoxy-Hb]**		**Δ[oxy-Hb]**		**Δ[t-Hb]**	
		**Mean**	**SD**	**Mean**	**SD**	**Mean**	**SD**
Left forehead	Oral	−0.327	0.838	−0.066	3.099	−0.392	3.053
	Written	0.022	0.785	2.580	2.635	2.603	3.075
	*P*	0.128		0.002^**^		0.001^**^	
Right forehead	Oral	−0.334	0.768	−0.061	3.437	−0.395	3.339
	Written	0.071	0.798	2.103	2.448	2.174	2.602
	*P*	0.068		0.012^*^		0.003^**^	
Full forehead	Oral	−0.330	0.756	−0.063	3.114	−0.393	2.981
	Written	0.047	0.752	2.341	2.437	2.388	2.747
	*P*	0.078		0.003^**^		<0.001^***^	

* *P < 0.05*;

** *P < 0.01*;

*** *P < 0.001*.

**Figure 2:**
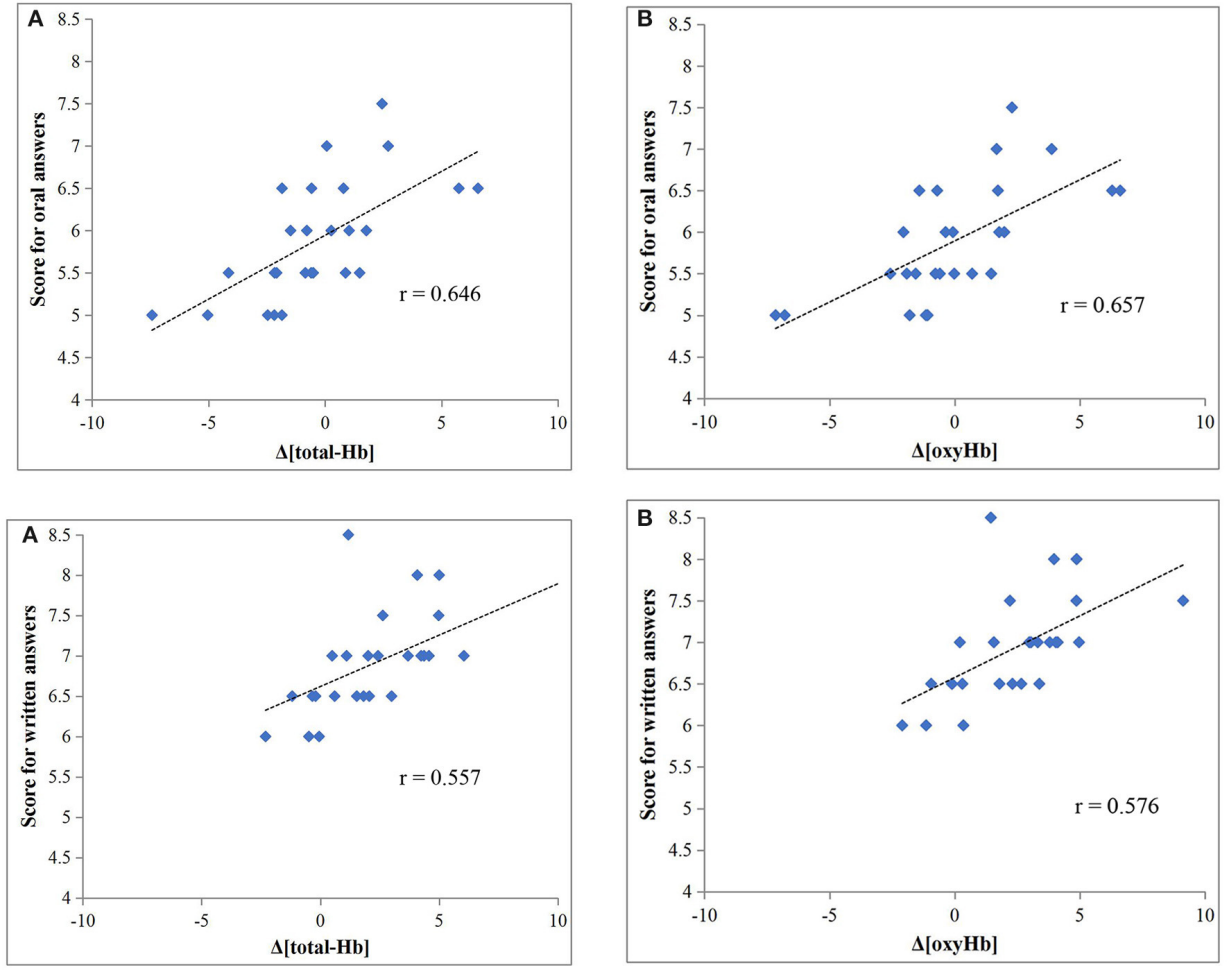
Relationship between test scores and Δ[total-Hb] **(A)** and Δ[oxyHb] **(B)**.

Both Δ[total-Hb] and Δ[oxyHb] were significantly correlated with scores for oral answers (*r* = 0.646 and *r* = 0.657, respectively; both *P* < 0.001) (Figures 2A,B), while there was no significant correlation with Δ[deoxyHb]. Both Δ[total-Hb] and Δ[oxyHb] were also significantly correlated with the scores for written answers (*r* = 0.557, *P* = 0.003 and *r* = 0.576, *P* = 0.002, respectively) (Figures 2A,B), and again there was no significant correlation with Δ[deoxyHb].

## Discussion

This study examined the hemodynamic responses of the mPFC during a short-term memory task and compared activation patterns between recall modalities (written vs. oral). The primary observations are that reading (acquisition) and recall (whether written or oral) can activate the mPFC, that response amplitude is greater during written recall, and that response magnitude is correlated with recall accuracy for both modalities.

Brain activation as measured by NIRS is associated with a regional increase in total hemoglobin concentration, usually consisting of a substantial increase in [oxyHb] and only a moderate increase in [deoxyHb] (Kubota et al., 2006). Compared to verbal responses, written answers were associated with greater increases in both [oxyHb] and [total-Hb], suggesting a greater cognitive load and ensuing metabolic demand on the mPFC during writing than verbalizing (Toichi et al., 2004; Petrides, 2005). In the Chinese education model, students are more likely to complete learning tasks through writing rather than talking, and these learning habits may have improved the subjects' capacity for solving problems in writing, thereby inducing more intense neural activity and brain metabolism (Asano et al., 2014). The change in [deoxyHb] is considered a sign of mPFC activation, and an increase in [deoxyHb] indicates an increase in mPFC oxygen consumption, which is associated with subjective effort in tasks requiring attention (Hoshi et al., 2003; Matsui et al., 2007; Ehlis et al., 2008). In this study, [deoxyHb] was moderately higher for subjects providing written responses, but the difference compared to oral responses did not reach a level of statistical significance. Nonetheless, this result suggests that the subjects were more subjectively focused while writing answers.

The accuracy of recall differs depending on presentation and recall modality. There are at least two plausible reasons for the greater mPFC activation while providing written responses. First, writing may more effectively stimulate memory loops involving the mPFC, MTL, and other cortical regions. Second, writing down ideas may promote inductive reasoning (i.e., brainstorming and sorting ideas) that may be more difficult to accomplish when providing an answer orally. It was apparent that many subjects had erased and/or changed their written answers.

Differences in body movement between the two recall modalities may also influence our results. In order to investigate whether the difference in mPFC activity is attributable to body movement, we randomly selected five subjects and measured their brain oxygen parameters when drawing circles and writing down past memories. We found that these actions displayed significantly different results (*P* < 0.05), indicating that mPFC activation is moreso correlated with memory recall rather than body movement.

During NIRS measurements, light is shone on the surface of the skin and is transmitted through the underlying tissue to a detector. Since human tissue is non-uniform, photons move randomly between the light source and the detector. To account for the additional pathlength traveled by near-infrared light, the differential pathlength factor is used (DPF) (Delpy et al., 1988; Scholkmann et al., 2018). DPF is related to the subject's age, the tissues in the photon's path, and various optical parameters. Due to equipment limitations, we did not account for the thickness of the subjects' forehead tissue and skull. In order to reduce the effect of the DPF, we selected younger adults (22–30 years old), all of whom are of Asian descent (similar skin tone) and from southern China. The subjects did not report any symptoms of anemia or hypertension, which would reduce the impact of bone thickness and differences in soft tissue on NIRS measurements (Yoshitani et al., 2007). Likewise, our experimental data is normalized to account for differences in brain oxygen parameters between participants.

Hemodynamic fluctuations on the surface of the scalp and forehead may lead to inaccurate results or false positives (Kirilina et al., 2012). To maintain hemodynamic stability, experiments were completed in relatively comfortable environments. The subject's blood oxygen level, blood pressure, heart rate, and forehead temperature were taken at three time points: (1) prior to the start of the experiment, (2) upon finishing the first article, and (3) upon finishing the second article. During our experiments, these measurements did not display significant changes. Thus, we are confident that because each subjects' hemodynamic parameters were fairly stable, the measured changes in cerebral oxygen parameters are correlated to brain activity.

Both [oxyHb] and [total-Hb] in mPFC were moderately correlated with higher recall scores, suggesting that these mPFC cerebral oxygen parameters might be predictive of subjective effort. Such NIRS-based measures may be particularly valuable for diagnosing and treating cognitive impairments (Yeung et al., 2016b) by revealing various aspects of executive function in real time.

## Limitations

We utilized a relatively small sample size drawn from a highly educated population. As such, the results presented may not be generalizable to the entire population. Likewise, due to the impact of acute sleep deprivation (<7 h) on working memory (Yeung et al., 2018), we did not include people who had slept <7 h. As such, our findings only reflect performance with adequate rest. We did not examine differences in acquisition modality or whether matching acquisition affects recall, so results may be related to the way information is obtained. In future studies, performance should be compared among multiple presentation modalities such as text, audio, and visual presentations. It is also of interest to assess the difficulty or amount of information provided and examine whether written answers are still more accurate than oral answers.

## Conclusion

Near-infrared spectroscopy can measure the degree of mPFC activation by detecting changes in cerebral oxygen parameters. The degree of mPFC activation differs according to recall modality when the input modality is held constant. Increases in [oxyHb] and [total-Hb] were greater during when providing answers in writing, and this enhanced mPFC activation was associated with greater response accuracy. mPFC oxygen parameters likely reflect the degree of exertion. From this, we observe that NIRS may be a valuable portable non-invasive modality for cognitive assessment in both healthy and patient populations.

## Data Availability Statement

The raw data supporting the conclusions of this article will be made available by the authors, without undue reservation.

## Ethics Statement

The studies involving human participants were reviewed and approved by Ethics Committee of Changzhou First People's Hospital. The patients/participants provided their written informed consent to participate in this study.

## Author Contributions

JZ helped with the conception and design, analysis, revising, and final approval of the article. YuZ helped with the interpretation of data, drafting, revising, and final approval of the article. YW and BL managed the randomization table and assigned the treatment groups and were in charge of data acquisition and statistical analyses. YiZ was the outcome assessor. All authors read and approved the final manuscript.

## Conflict of Interest

The authors declare that the research was conducted in the absence of any commercial or financial relationships that could be construed as a potential conflict of interest.
